# Effect of argon plasma and dentin rehydration on bond strength of dental adhesives to etched dentin

**DOI:** 10.1590/0103-644020246029

**Published:** 2024-12-16

**Authors:** Daniela Bandeira dos Santos, Vitaliano Gomes de Araújo, Amanda Endres Willers, Carolina Bosso André, Marcelo Giannini

**Affiliations:** 1 Department of Restorative Dentistry. Piracicaba Dental School, University of Campinas, Brazil; 2 Department of Restorative Dentistry. School of Dentistry, Federal University of Minas Gerais, Brazil

**Keywords:** Adhesives, Argon plasma, Microtensile bond strength, Dentin, Scanning electron microscopy

## Abstract

This study investigated the effects of nonthermal atmospheric pressure plasma (NAPP) application and dentin rehydration with water (REHY) on bond strength (BS) of adhesives. Three etch-and-rinse adhesives were tested: Scotchbond Multi-Purpose (SBM / water-based primer + adhesive resin), Gluma Bond Universal (GBU / single-bottle containing acetone as organic solvent) and Prime&Bond Universal (PBU / single-bottle containing propanol as organic solvent). Adhesives were applied: 1- to phosphoric acid-etched dentin (Control), 2- after NAPP application for 45 seconds to etched dentin or 3- after REHY with water (10 seconds) of plasma-treated etched dentin. BS was performed using human molars and microtensile test, in which specimens were tested after 24 hours or one year (n=6). BS data were analyzed by three-way mixed ANOVA and Bonferroni’s test (α=0.05). The morphology of etched dentin (n=3) and the dentin-adhesive interfaces (n=3) were evaluated using SEM. NAPP and REHY did not increase the BS of GBU and PBU. The BS of SBM to etched dentin not treated with NAPP (Control) reduced significantly after one year. The PBU adhesive showed the highest bond strength to dentin when the adhesives tested were applied after REHY. Phosphoric acid demineralized dentin to a depth of 5 mm that allowed the adhesive penetration into the dentinal tubules, forming the resin tags. Interfacial structures were maintained after one year. Argon plasma treatment was not beneficial in increasing the dentin bond strength of adhesives to etched-dentin at 24 hours. However, rehydration after plasma application prevented the bond strength reduction for three-step adhesive after one year.

## Resumo

Este estudo investigou os efeitos da aplicação de plasma não térmico à pressão atmosférica (PLAR) e da reidratação dentinária com água (REID) na resistência de união (RU) de adesivos. Foram testados três adesivos convencionais: Scotchbond Multi-Purpose (SBM / primer à base de água + resina adesiva), Gluma Bond Universal (GBU / frasco único contendo acetona como solvente orgânico) e Prime&Bond Universal (PBU / frasco único contendo propanol como solvente orgânico). Os adesivos foram aplicados: 1- na dentina condicionada com ácido fosfórico (Controle), 2- após aplicação de PLAR por 45 segundos na dentina condicionada ou 3- após REID com água (10 segundos) na dentina condicionada tratada com plasma. A RU foi realizada utilizando molares humanos e teste de microtração, em que as amostras foram testadas após 24 horas ou um ano (n=6). Os dados de RU foram analisados por ANOVA mista de três fatores e teste de Bonferroni (α=0,05). A morfologia da dentina condicionada (n=3) e as interfaces dentina-adesivo (n=3) foram avaliadas por MEV. PLAR e REID não aumentaram a RU da GBU e PBU. A RU do SBM para a dentina condicionada não tratada com NAPP (Controle) reduziu significativamente após um ano. O adesivo PBU apresentou maior resistência de união à dentina quando os adesivos testados foram aplicados após REID. O ácido fosfórico desmineralizou a dentina até uma profundidade de 5 mm que permitiu a penetração do adesivo nos túbulos dentinários, formando tags de resina. As estruturas interfaciais foram mantidas após um ano. O tratamento com plasma de argônio não foi benéfico no aumento da resistência de união dentinária dos adesivos à dentina condicionada em 24 horas. Entretanto, a reidratação após a aplicação do plasma evitou a redução da resistência de união do adesivo de três etapas após um ano.

## Introduction

Dentin acid etching with 30-40% phosphoric acid removes minerals from intra- and extra-fibrillar collagen portions at intertubular and peritubular dentin areas. In addition to superficial demineralization, the removal of the smear layer and smear plugs also occurs. The etched dentin presents opened dentinal tubules and increased permeability ^1^.

The main concerns related to bonding durability are still the structural stability of adhesive polymers, the difficulty in adhesive resin infiltration into demineralized dentin to complete filling the interfibrillar spaces and avoiding the enzymatic degradation of poorly infiltrated collagen fibrils that were exposed after acid etching ^2^. Some studies have shown that moisture conditions of acid-etched dentin can affect the quality of the hybrid layer formation and dentin bond strength for conventional, etch-&-rinse adhesives ^3^ ^,^ ^4^. The excessive drying of acid-etched dentin is inadequate because it leads to dentin desiccation with reduction of interfibrillar spaces and collapsed collagen fibrils, which does not allow the penetration of adhesive resin into interfibrillar spaces ^5^.

Plasma consists of partially ionized gases containing highly reactive particles, such as electronically excited atoms, molecules and free radical species ^6^. Because of its effects of increasing surface energy of materials without heat damage and change of topography, nonthermal atmospheric pressure plasma (NAPP) has been indicated in dental adhesion ^7^ ^,^ ^8^ ^,^ ^9^. Studies have shown benefits with the use of NAPP in the resin penetration of adhesives and in the polymerization of resinous materials ^6^ ^,^ ^7^ ^,^ ^10^ ^,^ ^11^ ^,^ ^12^.

However, NAPP application can dehydrate etched dentin and this lack of dentin moisture surface before the adhesive application can impair the adhesive monomer infiltration, because of the collapse of collagen fibrils caused by the plasma jet ^5^ ^,^ ^8^. This may be the reason for the non-increase in bond strength to dentin despite plasma-treated dentin, as observed by some studies ^12^ ^,^ ^13^. Theoretically, dehydration caused by NAPP applications could be overcome by using water to rehydrate etched dentin and promote the fibrillar re-expansion before adhesive application ^5^.

The aim of this study was to investigate the effects NAPP application and dentin rehydration (REHY) on bond strength of three adhesives. Additionally, the analysis of failure modes of tested specimens, the morphology of etched dentin and the morphology of dentin-adhesive interfaces were investigated. The research hypotheses were that: 1- the dentin rehydration with water after NAPP application increases the bond strength to etched dentin, regardless of the type of adhesive, and 2- the bond strength of adhesives to dentin treated with NAPP is not reduced after one year.

## Materials and Methods

### Teeth Preparation and Experimental Groups

Sixty-three sound human third molars were collected and used according to a protocol approved by the local research ethics committee (CAAE #56073422.9.0000.5418). All teeth were cleaned by hand scaling with a periodontal curette (SS White Duflex; Juiz de Fora, MG, Brazil), and polished with a paste of pumice and water. Afterwards, they were stored in thymol solution (Labsynth; Diadema, SP, Brazil) at 4ºC for no longer than three months.

Teeth were attached to an automated sectioning machine (Buehler Ltd., Lake Bluff, IL, USA).and their crowns and roots were removed using a diamond saw (Isomet Diamond Wafering Blade, Buehler Ltd, Lake Bluff, IL, USA). These sections were cut under running water. The teeth were sectioned 2 mm below the cemento-enamel junction to remove their roots, and 2 mm above the dentin-enamel junction to remove the occlusal enamel and expose the dentin surface at middle depth. Dentin surfaces were polished with a silicon carbide paper (600-grit) under water-cooling for 10 seconds, aiming to create a standardized smear layer.

Dentin was etched with 37% phosphoric acid gel (Dentsply Sirona, Pirassununga, SP, Brazil) for 15 seconds and rinsed for 20 seconds. After rinsing, the dentin remained with water on its surface. Excess water was removed with absorbent paper (Yuri, Manikraft, São Paulo, SP, Brazil) and the etched dentin remained moist for application of the adhesive ^4^. Teeth were randomly divided into three dentin treatments and adhesive applications groups:

- Control: adhesives were applied to phosphoric acid-etched and moist dentin (CONT).
- NAPP was applied for 45 seconds to phosphoric acid-etched and moist dentin (in each 16 mm^2^ region of the dentin surface) before adhesive application (NAPP).
- Phosphoric acid-etched and NAPP-treated dentin was rehydrated with distilled water for 10 seconds before adhesive application (REHY).

The plasma equipment used in this study (Surface Brasil - Atmospheric plasma equipment, Campinas, SP, Brazil) consisted of a hand-held unit (130 mm length x 30 mm diameter), with a quartz nozzle (4 mm length x 2 mm diameter) attached to a high voltage power supply used to produce a non-thermal plasma torch at atmospheric pressure ^12^. High purity argon gas (Praxair 4.8; White Martins, Rio de Janeiro, RJ, Brazil) was used at 1 liter per minute to produce a plasma torch. The cold thermal “torch” exiting the nozzle was 10 mm long x 2 mm diameter and operated at room temperature of 23^o^ C. The distance between plasma-jet nozzle end and the dentin surface was 5 mm, with the hand-held unit placed vertically.

NAPP was applied to moist dentin for 45 seconds in each 16 mm^2^ region of the dentin surface. Depending on the occlusal dentin surface area of each tooth, five to six different regions of the etched dentin surface were treated with NAPP, in order to cover the entire dentin surface. CONT received no NAPP treatment.

Rehydration of plasma-treated dentin consisted of applying 40 microliters of water to the dentin surface prior to application of the adhesive. The water bubble remained on the dentin surface for 10 seconds, when it was removed with absorbent paper and the dentin remained moist for adhesive application.

Three commercial dental adhesives were tested in this study (etch-and-rinse type): a water-based primer and hydrophobic adhesive resin (Scotchbond Multi-Purpose, 3M Oral Care, St. Paul, MN, USA) (SBM); an acetone-based adhesive (Gluma Bond Universal, Kulzer - Mitsui Chemicals Group, Hanau, Germany) (GBU) and an isopropyl alcohol-based adhesive (Prime&Bond Universal, Dentsply Sirona, Konstanz, Germany) (PBU). Compositions, application techniques and batch numbers of adhesive systems tested in this study are described in Box 1.

**Box 1:**
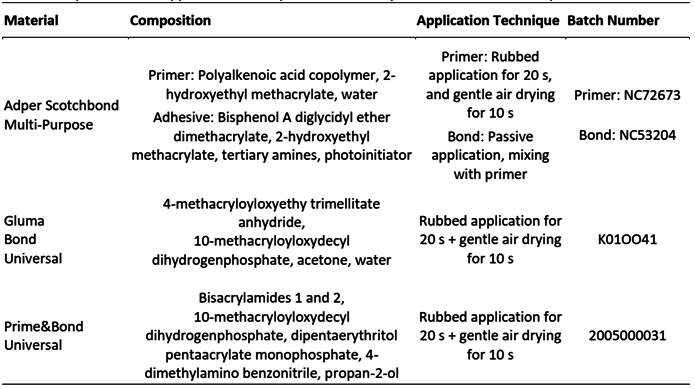
Compositions and application techniques of adhesive systems tested in this study.

### Dentin Bond Strength Test

Fifty-four third molars were used (n = 6) ^14^ in this part of the study. After the adhesive application and its light-activation for 10 seconds (1,050 mW/cm^2^, Valo Cordless, Ultradent products Inc., South Jordan, UT, USA) and a block of composite was incrementally built-up over bonded dentin using 2-mm thick for each composite layer (Charisma Classic, Kulzer, Hanau, Germany) that were light-cured for 20 seconds each layer.

Teeth were stored in water for 24 hours at 37^o^C. Afterwards, they were serially sectioned in buccal-lingual and mesial-distal directions using a diamond saw (Buehler Ltd, Lake Bluff, IL, USA) that was attached to a sectioning machine (Buehler Ltd., Lake Bluff, IL, USA) to obtain 8 to 12 specimens (or “sticks”) per tooth with a cross section area of approximately 1.0 mm^2^. Half of the specimens (4 to 6 specimens) from each tooth were immediately tested, while the other half (4 to 6 specimens) was tested after storage for one year in distilled water at 37°C. Distilled water was replaced monthly.

The specimens were tested on a microtensile device attached to a universal testing machine (EZ Test, Shimadzu, Kyoto, Japan). Each stick was fixed to the device using cyanoacrylate-based glue (Super Bonder Gel, Henkel/Loctite, Diadema, SP, Brazil), and tested at a constant speed of 0.5 mm/min until failure of the bonding interface. The peak tensile load obtained after testing each specimen was divided by the cross-sectional area to calculate the bond strength (in MPa). The bond strength values for 4 to 6 specimens from the same tooth were used to calculate the bond strength mean for each tooth at both times.

### Failure Mode Analysis

After bond strength test, the fractured surfaces of tested specimens were sputter coated with gold (MED 010, Balzers, Balzer, Liechtenstein) and examined using a scanning electron microscope (JSM-IT300, Jeol Inc., Tokyo, Japan) at 100x magnification (voltage: 15 kV; beam width: 25-30 nm; working distance: 10-20 mm). The fractures were classified as the following failure modes:

Type I: cohesive failure within resin composite;

Type II: adhesive failure between composite resin and adhesive;

Type III: Adhesive failure between the dentin surface and adhesive;

Type IV: Mixed failure characterized by the exposure of the dentin surface, the adhesive and/or the presence of the restorative material;

Type V: cohesive failure within the adhesive layer;

Type VI: cohesive failure within dentin;

Type VII: cohesive failure within the hybrid layer.

### Micromorphological Analysis of the Etched Dentin

For this part of the study, nine teeth were used (n = 3). The teeth were prepared for the bond strength test and their dentin surface was etched with phosphoric acid for 15 seconds (CONT), treated with NAPP or REHY. A notch was created on the mesial and distal surfaces of the teeth to fracture the dental crown after phosphoric acid etching and fixation with 2.5% glutaraldehyde for 12 hours, followed by immersion in a 0.2 M phosphate-buffered saline solution (pH 6) for 1 hour, which was changed each 20 minutes. A chisel was used to cause mesiodistal fracture.

Samples were dehydrated for 20 minutes in each one of the following ethanol solutions: 25%, 50%, 75%, 90%, and absolute ethanol. A final dehydration process by means of critical-point drying was carried out (CPD 030 critical point dryer, Bal-Tec AG, Balzers, Liechtenstein), and the dehydrated samples were sputter-coated with gold (MED 010, Bal-Tec AG, Balzers, Liechtenstein) to be observed using a scanning electron microscope (JSM-IT300, Jeol Inc., Tokyo, Japan) at 10,000x magnification.

### Micromorphological Analysis of the Dentin-Adhesive Interface

Three specimens from the experimental group and controls not used for bond strength test were prepared for micro-morphological interfacial characterization. The specimens were embedded in epoxy resin (EpoxiCure 2 system; Buehler Ltd., Lake Bluff, IL, USA) and ground with a sequence of SiC papers (600-, 1,200-, and 2,000-grit), followed by polishing using diamond pastes (6, 3, and 1 μm). They were subsequently thoroughly rinsed with water and the remaining polishing debris were removed by 5-minutes ultrasonic clean. After polishing, specimens were etched with 50% phosphoric acid for 15 seconds, rinsed, treated with 0.1% sodium hypochlorite for 10 minutes, and rinsed with water.

Specimens were dehydrated for 10 minutes in each one of the following ethanol solutions: 25%, 50%, 75%, 95%, and absolute ethanol. Embedded specimens were overnight dried (at 37°C), mounted on aluminum stubs and sputter coated with gold, before the evaluation under electron microscopic evaluation (JSM-IT300, Jeol Inc., Tokyo, Japan). Representative areas of the dentin-adhesive interfaces of experimental groups and controls were photographed at 1,000x magnification.

### Statistical Analysis

Data distributions were verified according to their normality, homoscedasticity, and sphericity. Data were transformed with square root and statistically analyzed by three-way analysis of variance (Mixed ANOVA, considering “Evaluation Time” a repeated factor / “Type of adhesive”, “Dentin treatment” and “Evaluation time” factors) and post-hoc Bonferroni’s test (α = 0.05). Statistical test was performed using the software SPSS Statistics (Version 23, IBM Corp, Armonk, NY, USA).

The specimen failure results were described according to the frequency of occurrence of the seven types of fractures for each group (dentin treatment and adhesive) and for both storage times (24 hours and one year). The morphological aspects of the etched dentin structures (plus after NAPP and REHY) and the dentin-adhesive interfaces for the experimental groups and controls observed in electron microscopy analyzes were reported.

### Results

### Out Dentin Bond Strength Test

Bond strength means are presented in Table 1. ANOVA showed that bond strength results were influenced significantly by the “type of adhesive” (p < 0.0001) and by the “evaluation time” (p < 0.0001) factors, but not by “dentin treatment” (p = 0.752). The interactions among factors were not significant (p < 0.05).

**Table 1:** Dentin bond strength means (SD), according to the adhesives, dentin treatments (CONT, NAPP and REHY) and evaluation times (24 hours and one year) (in MPa).

	Adhesive	CONT	NAPP	REHY
24 h	Scotchbond Multi-Purpose	47.5 (8.1) ab A	44.9 (5.6) a A	43.5 (8.3) b A
	Gluma Bond Universal	39.1 (5.6) b A	44.9 (7.6) a A	42.6 (4.7) b A
	Prime&Bond Universal	54.6 (8.8) a A	54.1 (8.7) a A	61.5 (5.3) a A
1 year	Scotchbond Multi-Purpose	38.7 (6.0) b A*	38.6 (4.5) a A	38.4 (12.9) b A
	Gluma Bond Universal	39.1 (7.9) ab A	41.5 (11.4) a A	39.4 (9.2) b A
	Prime&Bond Universal	52.4 (9.4) a A	48.2 (3.0) a A	54.6 (9.6) a A

Different letters indicate significant difference, according to the Mixed ANOVA and post-hoc Bonferroni’s test (α = 0.05).

Lower case letters compare adhesives within the same dentin treatment and evaluation time.

Upper case letters compare dentin treatments for the same adhesive and evaluation time.

(*) Compare 24 hours and one year, indicating significant difference from 24 hours for the same adhesive and dentin treatment.

For the CONT, PBU adhesive showed higher dentin bond strength than those obtained for GBU and SBM adhesives at 24 hours and one year, respectively. On the other hand, PBU exhibited bond strength values statistically similar to the SBM adhesive at 24 hours and the GBU adhesive at one year.

The application of NAPP to etched dentin yielded no significant bond strength difference among adhesives. For the REHY treatment, the bond strength of PBU was higher than SBM and GBU, regardless of the evaluation time.

The NAPP and REHY dentin treatments did not significantly increase the dentin bond strength, regardless of the type of adhesive and evaluation time. However, the bond strength to dentin reduced significantly after one year only for SBM, when NAPP was not applied to dentin.

### Failure Mode Analysis

**Figure 1:**
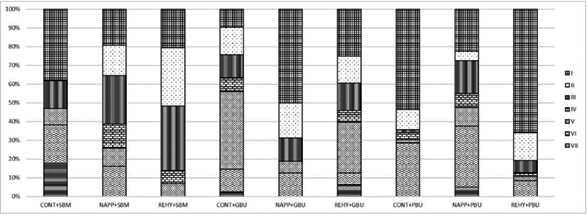
Bar graph showing the percentage of failure modes for all groups at 24 hours.

**Figure 2:**
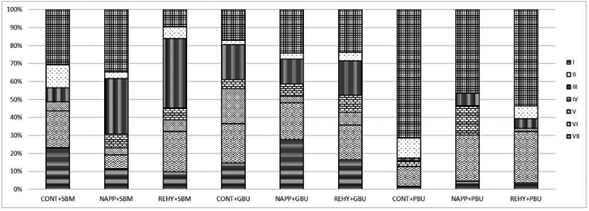
Bar graph showing the percentage of failure modes for all groups at one year.

Only failure types I, III and V occurred for all groups at 24-hour (Figure 1), while the failure types I, II, III, VI and VII were obtained for all groups at one year (Figure 2). In general, an increase in type VII failure (cohesive failure within hybrid layer) was observed for SBM at one year. Also, the incidence of type III failure (adhesive failure between dentin and adhesive) increased for the NAPP and REHY treatment groups of SBM. For GBU, the percentage of type III failure (adhesive failure between dentin and adhesive), type VI (cohesive failure within dentin) and type VII (cohesive failure within hybrid layer) increased at one year. For PBU, the type I failure (cohesive failure within composite) increased for CONT and NAPP treatments at one year.

### Micromorphological Analysis of the Etched Dentin and Dentin-Adhesive Interfaces

**Figure 3:**
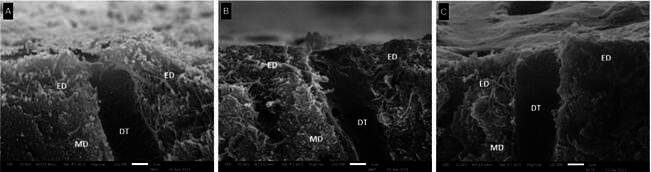
Representative scanning electron microscopy micrographs of cross section of etched-dentin (Figure A), treated with NAPP (Figure B) and REHY (Figure C). Abbreviation: ED- etched dentin, MD- mineralized dentin and DT- dentin tubule.

**Figure 4:**
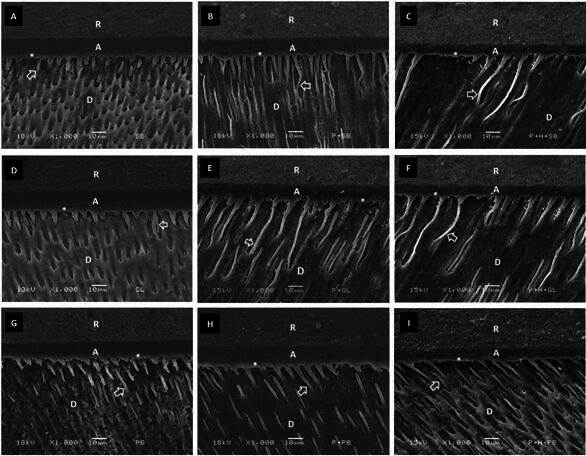
Representative scanning electron microscopy micrographs of the adhesive-dentin interfaces at 24 hours, according to the dentin treatments (CONT, NAPP and REHY). (A) CONT + SBM; (B) NAPP + SBM; (C) REHY + SBM; (D) CONT + GBU; (E) NAPP + GBU; (F) REHY + GBU; (G) CONT + PBU; (H) NAPP + PBU; (I) REHY + PBU. Abbreviations: R- composite resin; A- adhesive layer; D- dentin. Arrows indicate resin tags and asterisks indicate the hybrid layer.

**Figure 5:**
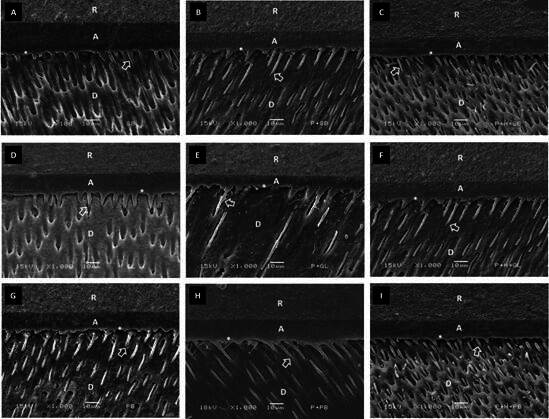
Representative scanning electron microscopy micrographs of the adhesive-dentin interfaces at one year, according to the dentin treatments (CONT, NAPP and REHY). (A) CONT + SBM; (B) NAPP + SBM; (C) REHY + SBM; (D) CONT + GBU; (E) NAPP + GBU; (F) REHY + GBU; (G) CONT + PBU; (H) NAPP + PBU; (I) REHY + PBU. Abbreviations: R- composite resin; A- adhesive layer; D- dentin. Arrows indicate resin tags and asterisks indicate the hybrid layer.

Figure 3 shows the dentin sub-surface etched with phosphoric acid, which demineralized the dentin to a depth ranging from 2 to 5 μm. Exposed collagen fibrils can be seen in this demineralized region. Figures 4 and 5 show the dentin-adhesive interfaces for all adhesives and dentin treatments. Phosphoric acid etching allowed the formation of hybrid layer and resin tags into the dentinal tubules for all groups (CONT, NAPP and REHY). Deeper penetration of the adhesives into the dentinal tubules was observed with the application of NAPP and REHY mainly at 24 hours.

## Discussion

REHY neither increased nor reduced the dentin bond strengths of adhesives compared to the CONT and NAPP, thus the first research hypothesis was rejected. On the other hand, the second research hypothesis was accepted, because the dentin bond strength of the three adhesives following the NAPP or REHY dentin treatments was not reduced after one year of water storage.

The SBM adhesive applied without previous NAPP treatment was the only group (CONT) that the dentin bond strength reduced at one year. It was observed the increase of failures within the hybrid layer (type VII) after one year of water storage, maybe due to the high hydrophilicity characteristic of SBM. Also, this type of adhesive has a primer containing polyalkenoic acid copolymer, HEMA monomer and water that may have difficulty penetrating the collagen fibril network of demineralized dentin ^3^ ^,^ ^15^. The NAPP application and the REHY kept SBM dentin bond strength stable after one year of water storage, showing the beneficial effects of NAPP application to etched dentin ^8^ ^,^ ^9^ ^,^ ^11^. The water can re-expand the collagen fibrils after the effects of dentin drying with NAPP application and the increase in “collagen fibrils reactivity” by NAPP application can improve the penetration of adhesive monomer into etched dentin ^7^ ^,^ ^8^ ^,^ ^13^, which contributed to maintaining the dentin bond strength stable after one year.

The permeability of the bonding monomer can be improved by replacing water with an alcohol or acetone solvent in the bonding resin, ensuring that the bonding monomer penetrates into the deepest parts of the demineralized layer to suppress the formation of remaining demineralized dentin ^3^. In this study, adhesives containing acetone (GBU) and propanol (PBU) as organic solvents did not yield increased bond strength when dentin was treated with NAPP or REHY. Studies showed that acetone, methanol, ethanol, propanol and glutaraldehyde increase the modulus of elasticity of demineralized dentin matrix and the stiffening rate was higher for acetone and ethanol (0.8 to 0.9 MPa/min) than methanol and propanol (0.5 to 0,6 MPa/min), but the increases in modulus were rapidly reversed by rehydration in water ^16^ ^,^ ^17^. The stiffness of wet demineralized human dentin matrix is very low, while the glutaraldehyde increases the demineralized dentin matrix stiffness, but it is not reversible by rehydration in water. As organic solvents quickly stiffen the demineralized dentin, it may be important in maintaining the dentin matrix expanded during the adhesive monomer infiltration and solvent evaporation ^16^ ^,^ ^17^.

GBU contains 4-META and 10-MDP adhesive promoter monomers and acetone and water as solvents. Both adhesive monomers (4-META and 10-MDP) form ionic bonds with calcium from hydroxyapatite that are important to obtain a durable bond strength ^18^. The bond strength of this universal adhesive to etched dentin did not differ significantly from the SBM, regardless of the type of dentin treatment and evaluation time. The bond strength of GBU to etched dentin was lower than that for PBU when testing the CONT and REHY dentin treatments at 24 hours and for REHY at one year.

According to the manufacturer, PBU can be applied to dry or wet etched dentin, because its formulation ensures adhesive monomer coverage of etched dentin and penetration at varying moisture levels by the well balanced hydrophobic and hydrophilic parts. In this study, the application of PBU after NAPP corresponded to dry dentin condition ^5^, while the wet condition was the Control. For the REHY moisture condition, PBU showed higher dentin bond strength than those obtained by SBM and GBU adhesives. The different dentin conditions (CONT, NAPP and REHY) did not influence the bond strength results for PBU, as observed for other adhesives. PBU contains two functional monomers (10-MDP and PENTA) and bisacrylamides in a solution of propanol that overcame the moisture conditions of this study.

Even using the critical point drying technique for observing samples in the scanning electron microscopy, it was not possible to observe differences in terms of fibrillar re-expansion with water after the application of NAPP. The dentin demineralized by phosphoric acid was not deeper than 5 μm, in which the hybrid layer was formed. The same structures observed for dentin-adhesive interfaces, regardless of the type of adhesive used, may explain the lack of influence of dentin treatments (CONT, NAPP and REHY) on bond strength to dentin. Greater penetration of the adhesives into the dentinal tubules was observed with the application of NAPP or REHY, as was also observed by previous studies ^7^ ^,^ ^10^ ^,^ ^13^. which is due to the surface energy increase of the NAPP treated dentin ^5^ ^,^ ^6^ ^,^ ^9^ ^,^ ^11^.

A systematic review and meta-analysis investigated the effects of NAPP application on dentin adhesion and reported that NAPP improved dentin wetting and bond strength with 30 seconds exposure time and up to 10 mm tip-to-surface distances ^19^. The plasma application protocol used in this study was slightly different (45 seconds of exposure and a distance of 5 mm) and the results did not show an increase in bond strength to NAPP treated-dentin. Studies have used different types of NAPP generating equipment, in which plasma is applied using different types of protocols. These variations may explain the different results obtained by these studies ^7^ ^,^ ^8^ ^,^ ^9^ ^,^ ^10^ ^,^ ^12^ ^,^ ^13^ and limit comparisons among them.

A study used a plasma jet system that was applied to the samples from a distance of 5 mm for 30 seconds and the power supply was operated at 5 liters/minute argon gas flow, 7 kV voltage, and 50 kHz frequency at room temperature ^7^. The plasma torch used by Kim et al. ^8^ was generated at a pulsed power of 0.3 Watts and the helium was the working gas that was delivered at a flow rate of 2 liters/minute. Another study used a plasma jet generated by helium gas, with a pulsing head of 2 GHz, 2 Pascal pressure, 2 Watts power and the application was performed in a closed chamber for 30 seconds with 5 seconds intervals by maintaining a 5 mm distance from the tooth surface ^9^. A commercial plasma generator consists of a hand-held unit with a pin-type electrode (1 mm diameter) surrounded by a 1.6-mm quartz capillary. The operating gas is argon, applied at a flow rate of 5 liters/minute and the plasma torch emerging at the exit nozzle is about 1.5 mm in diameter ^10^ ^,^ ^13^.

The protocol of NAPP application used in this study yielded an important clinical significance, which was the beneficial effect of NAPP on dentin bond strength stability for one year. The application of NAPP to dentin and the rehydration of dentin with water after NAPP application did not influence the bond strength of Gluma Bond Universal and Prime&Bond Universal adhesives. A study showed that the degree of dentin moisture did not seem to affect the clinical performance of a simplified etch-and-rinse adhesive in class I and II restorations that involved adhesion to enamel and dentin ^20^. In this study only dentin adhesion was evaluated, and the bond strength of Scotchbond Multi-Purpose was not reduced after one year when dentin was treated with NAPP. Prime&Bond Universal adhesive showed the highest bond strength among tested adhesives, when dentin was treated with phosphoric acid, NAPP and rehydrated with water.
